# CLIMATE CHANGE AND OLDER PEOPLE

**DOI:** 10.1093/geroni/igae098.1005

**Published:** 2024-12-31

**Authors:** Paola Zaninotto, Liat Ayalon

**Affiliations:** Chair; Co-Chair

## Abstract

The symposium focuses on the intersection of climate change impacts and the aging population. It includes presentations on the unique vulnerabilities of older adults to climate-induced disasters, the effect of heat exposure on their immune functions, the implications of extreme temperatures on healthcare utilization among the elderly in Europe, insights from older adults on climate change through a photovoice study, and strategies for building resilient communities for older adults. Each presentation would draw from comprehensive research and findings to highlight the need for targeted interventions and policies that address the specific challenges faced by older populations in the context of a changing climate. Climate Change and Aging Interest Group Sponsored Symposium

